# The Topographical Optimization of 3D Microgroove Pattern Intervals for Ligamentous Cell Orientations: In Vitro

**DOI:** 10.3390/ijms21249358

**Published:** 2020-12-08

**Authors:** Min Guk Kim, Chan Ho Park

**Affiliations:** 1 Department of Dental Science, Graduate School, Kyungpook National University, Daegu 41940, Korea; minguk.kim@knu.ac.kr; 2 Department of Dental Biomaterials, School of Dentistry, Kyungpook National University, Daegu 41940, Korea; 3 Institute for Biomaterials Research and Development, Kyungpook National University, Daegu 41940, Korea

**Keywords:** periodontal ligament, 3D printing, biopolymer, tissue engineering, microgroove, topography, optimization, regenerative medicine, cell alignment

## Abstract

Specific orientations of periodontal ligaments (PDLs) to tooth-root surface play an important role in offering positional stabilities of teeth, transmitting and absorbing various stresses under masticatory/occlusal loading conditions, or promoting tissue remodeling by mechanical stimulations to periodontal cells. However, it is still challenging to spatially control PDL orientations and collective PDL cell alignments using 3D scaffold architectures. Here, we investigated the optimization of scaffold topographies in order to control orientations of human PDL cells with predictability in in vitro. The 3D PDL-guiding architectures were designed by computer-aided design (CAD) and microgroove patterns on the scaffold surfaces were created with four different slice intervals such as 25.40 µm (μG-25), 19.05 µm (μG-19), 12.70 µm (μG-12), and 6.35 µm (μG-6) by the digital slicing step. After scaffold design and 3D wax printing, poly-ε-caprolactone (PCL) was casted into 3D printed molds and human PDL cells were cultured for 7 days. In the results, μG-25 with low vertical resolution can angularly organize seeded cells predictably rather than μG-6 created by the highest resolution for high surface quality (or smooth surface). Moreover, nuclear orientations and deformability were quantitatively analyzed and a significant correlation between microgroove pattern intervals and cell alignments was calculated for the topographic optimization. In conclusion, controllable microgroove intervals can specifically organize human PDL cells by 3D printing, which can create various surface topographies with structural consistence. The optimal surface topography (μG-25) can angularly guide human PDL cells, but 6.35 µm-thick patterns (μG-6) showed random organization of cell collectivity.

## 1. Introduction

Periodontal ligaments (PDLs) are the fibrous connective tissues between alveolar bone and cementum on tooth-root surfaces and have heterogeneous cell populations, which are characterized for osteogenic or cementogenic differentiation, immunogenic responses, or proliferation of progenitive PDL cells [1,2,3]. The collagenous Sharpey’s fibers at the terminal portions of PDL bundles should be inserted into the socket surfaces of alveolar bone and the cementum layers to form the hierarchical constructs with tissue integrations as the periodontal complex and the alveolar bone-PDL-cementum [4,5,6]. In particular, PDL fibrous bundles have specific orientations based on regions within 250–300 μm PDL interfaces and play a critical role in generating biomechanical responses against mastication [7,8]. Oblique or perpendicular orientations of PDLs to natural tooth-root surfaces transmit mastication forces, generate biological or physiological responses with biomechanical senses, or adapt to tooth movements via alveolar bone remodeling process [8,9].

Periodontitis, which is a chronic inflammatory disease, results in the destruction of mineralized tissues and connective tissue attachments supporting tooth structures [10,11,12]. Many studies have mainly investigated how to promote alveolar bone regeneration for dental implants or prosthetics after tooth extraction and bone graft material implantation [13,14]. However, it is still challenging to regenerate tooth-supporting tissues, especially from engineered PDLs with spatiotemporal controls of specific fiber orientations in micron-scaled PDL interfaces against ankylosis, which is the bone fusion to tooth-root surface by the excessive bone growth [1,15,16]. Of various technologies like soft lithography process [15,17] or freeze-casting fabrication [8], 3D printing can manufacture 3D scaffolds based on the computer-designed models for multiple periodontal tissue regeneration, such as alveolar bone, PDLs, and cementum [16,18]. In the previous study, 3D printing could create microgroove patterns on surfaces of PDL-guiding architectures with different angulations (0°, 45°, 90°) to the reference direction [16]. Interestingly, angular orientations of human PDL cells were regulated by the topographical microgroove angles and validated with anisotropic nuclear morphologies, which could characterize topographical interactions between material surfaces and cells [16,19,20]. Although angulated topographies facilitated to derive collective cell orientations and modulate specific cell organizations with high predictability [16], it is required to optimize surface topographies for controllable alignments of cells. In this follow-up study, we investigated how microgroove pattern intervals on surfaces of PDL-guiding architectures were configured with four different layer thicknesses at the digital slicing step to characterize anisotropic morphologies of cells and re-organize cytoskeletal components following patterns.

## 2. Results

### 2.1. Morphological Analyses of PDL-Guiding Architectures

**Figure 1:**
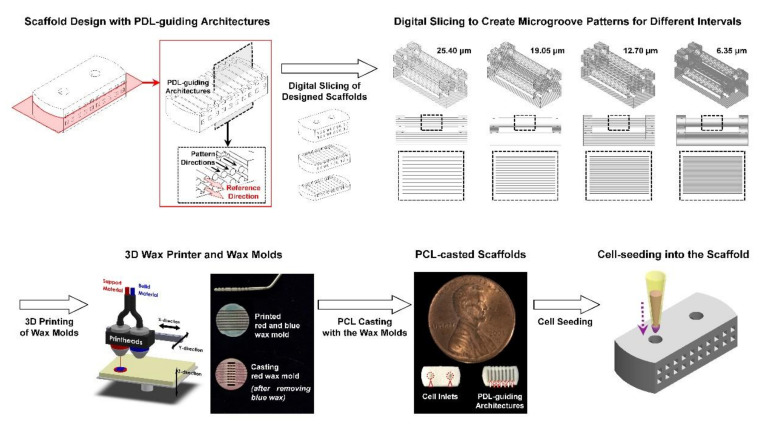
Four different scaffolds were designed with four different microgroove pattern intervals during the digital slicing step. After setting layer-by-layer thickness (25.40 µm, 19.05 µm, 12.70 µm, and 6.35 µm), wax molds were additively manufactured and poly-ε-caprolactone (PCL) scaffolds were casted. For investigation of in vitro cell alignments, human periodontal ligament (PDL) cells were seeded into the scaffolds for 7-day cultivation.

After the scaffold design and digital slicing steps, the 3D wax printer additively manufactured casting wax molds with the layer thickness (or layer-by-layer intervals) of the process set-up: 25.40 µm (μG-25), 19.05 µm (μG-19), 12.70 µm (μG-12), and 6.35 µm (μG-6) (Figure 1).

**Figure 2:**
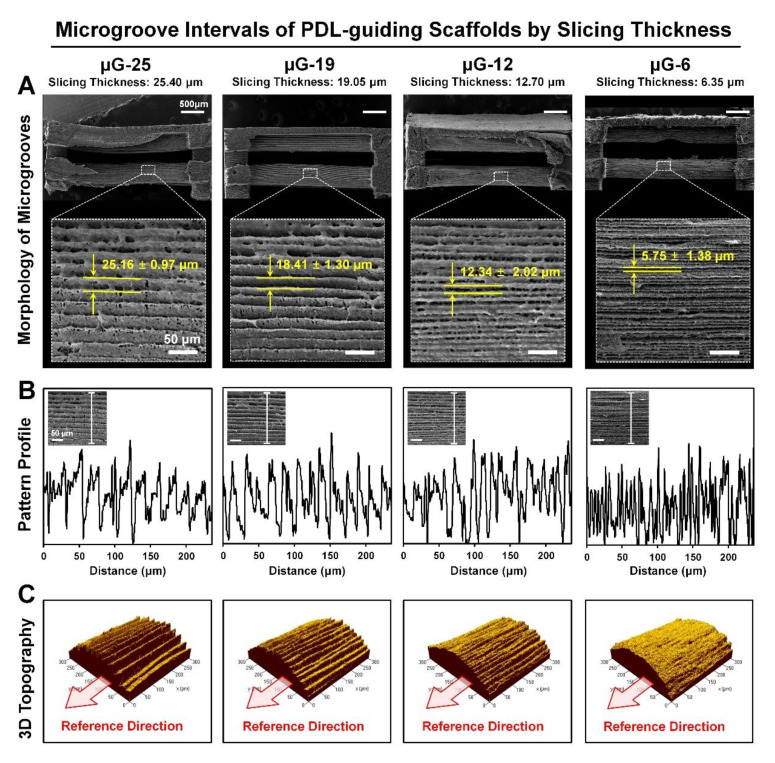
Qualitative and quantitative assessments to identify different of microgroove pattern intervals. (**A**) Scanning electron microscope (SEM) showed surface morphologies with the statistically calculated microgroove intervals in four different groups (μG-25, μG-19, μG-12, and μG-6) by different slice thickness. (**B**) Based on the SEM images, microgroove patterns were quantitatively analyzed and profiled by crossing the pattern surfaces on PDL-guiding architectures. (**C**) Using the confocal microscope, surface topographies were characterized, as was surface roughness.

Four different scaffold molds with four types of pattern intervals were casted using 25% poly-ε-caprolactone (PCL) biopolymer in 1,4-dioxane and scaffolds were quantitatively and qualitatively analyzed by the scanning electron microscopic (SEM) (Figure 2A). The layer-by-layer microgroove intervals were linearly measured and statistically resulted in 25.16 ± 0.97 μm (mean ± standard deviation; μG-25), 18.41 ± 1.30 μm (μG-19), 12.34 ± 2.02 μm (μG-12), and 5.75 ± 1.38 μm (μG-6) (Figure 2B). Moreover, 3D topographical images by the confocal microscope showed spatial morphological of pattern structures on PDL-guiding architectures (Figure 2C). Therefore, digital configurations at the digital slicing step can be the key player to modulate the microgroove patterns and sufficiently reflect the topographies of manufactured features with the significant correlations determined by surface morphology and 3D topography assessments (Figure 2).

### 2.2. Quantification of Collective Cell Orientations for Microgroove Optimizations

**Figure 3:**
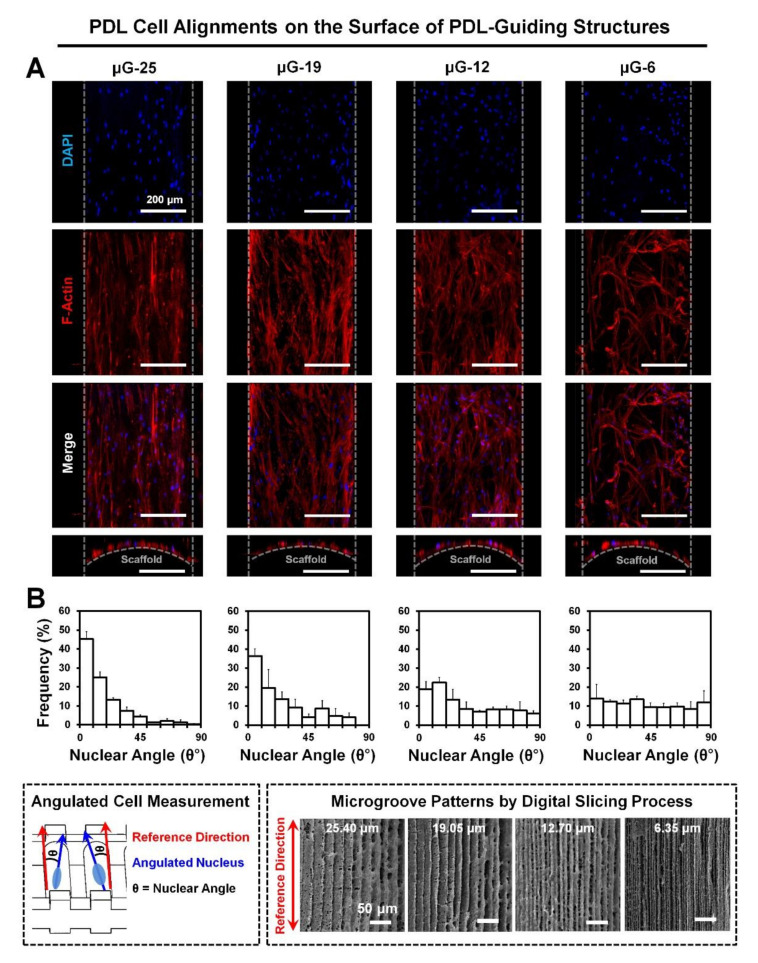
Cell orientations and angulations were analyzed using the fluorescence staining method with cell nuclei and actin filaments. (**A**) Cytoskeleton polarities or cell orientations to the reference direction can be qualitatively determined based on the actin filament staining for 7-day cultures. (**B**) Nuclear angulations were measured using directionalities of the 4′,6-diamidino-2-phenylindole (DAPI) stained cell nuclei and distributions of nuclei were defined with the statistical quantification. White dash-lines represent the ligament architecture border lines with a 250-μm distance. Scale bars: 200 μm.

A total of 1.0 × 10^3^ human PDL cells were seeded in a single scaffold and cultured for seven days. To analyze angular organizations of human PDL cells on the PDL-guiding architectures of four different microgroove interval groups, fluorescence-staining methods for nuclei (DAPI) and actin filaments (F-actin) were performed (Figure 3A). Cell orientations and alignments on PDL-guiding structures were quantified using angles of DAPI-stained nuclei of human PDL cells and measured with the reference direction as the previous studies described [16,18]. In general, the orientation of a single cell was measured and analyzed using the polarized cytoskeleton of a cell, arranged microfilaments in the cytoplasm, or deformed nuclear shapes, which were interactive responses to substrates [16,21].

The typical number (or frequency) of angulated nuclei (0–20°) were calculated and remarkable distributions of individual intervals of microgroove patterns showed in Figure 3B. In the results, alignments of approximately 70% cell nuclei corresponded to the microgroove patterns with 25.40-μm-intervals (μG-25) with high predictability, but the number of aligned cell nuclei, ranging in angle from 0 to 20°, decreased as intervals between pattern ridges were closer: frequencies of aligned nuclei = 70.26 ± 2.49% in μG-25, 55.84 ± 12.67% in μG-19, 41.27 ± 1.33% in μG-12, and 26.34 ± 8.16% in μG-6 (Figure 3B).

### 2.3. Quantification Assessments of Nuclear Deformation

**Figure 4:**
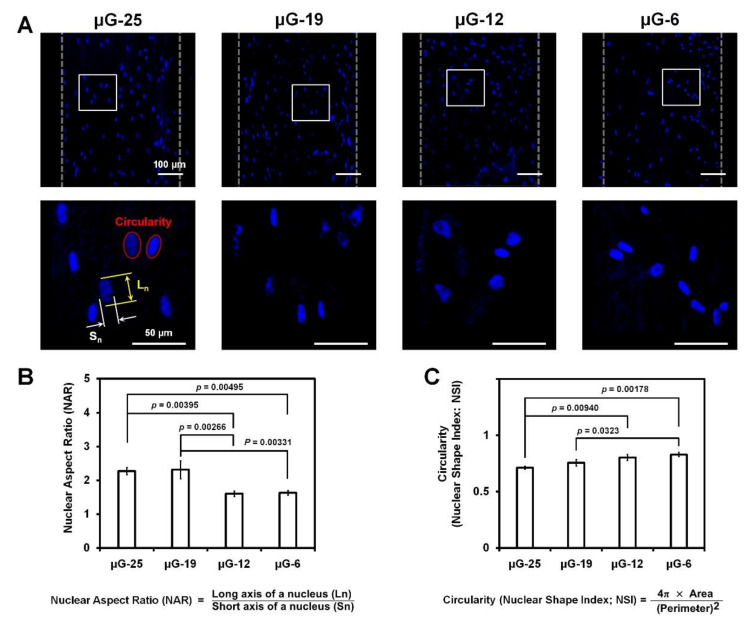
The nuclear aspect ratio (NAR) and circularity (or nuclear shape index; NSI) were measured and calculated to determine the deformation of cell nuclei. (**A**) DAPI-stained cell nuclei showed qualitative correlations of the nuclear deformations with four different microgroove pattern intervals (25.06 μm layer thickness for μG-25, 19.06 μm for μG-19, 12.70 μm for μG-12, and 6.35 μm for μG-6). (**B**) The NAR showed that μG-25 and μG-19 had more nuclear elongation than μG-12 and μG-6 with quantitative analyses and statistical calculations. (**C**) Circularity (or NSI) values were determined nuclear deformations by the perimeters of nuclei and quantitatively assessed with statistical calculations. Scale bars: 100 μm.

To validate the significant correlation between nuclear shapes and surface topographies, the nuclear aspect ratio (NAR) and the circularity (or nuclear shape index; NSI) of cell nuclei were qualitatively and quantitatively analyzed with statistics (Figure 4).

The quantification of NAR resulted that NAR_μG-25_ = 2.27 ± 0.11 and NAR_μG-19_ = 2.31 ± 0.26 groups had significantly more deformed nuclei than NAR_μG-12_ = 1.60 ± 0.077 and NAR_μG-6_ = 1.63 ± 0.065 and circularity (or NSI) showed that types of microgroove intervals on 3D substrates could morphologically affect circular shapes of nuclei such as Circularity_μG-25_ = 0.71 ± 0.012, Circularity_μG-19_ = 0.76 ± 0.029, Circularity_μG-12_ = 0.80 ± 0.027, and Circularity_μG-6_ = 0.83 ± 0.020 with statistically significant differences; *p*-value (NAR) = 0.000455 and *p*-value (circularity) = 0.00139 in ANOVA (Figure 4). Moreover, Bonferroni post-hoc analyses of NAR and circularity were calculated and μG-25 and μG-19 had statistically significant differences from smooth surface architecture groups (μG-12 and μG-6; Figure 4B,C) even though μG-25 and μG-19 showed no difference. Therefore, microgroove pattern intervals could influence cell orientations, nuclear deformation, and cell elongation, which were assessed by nuclear angulation, circularity (or NSI), and NAR, respectively.

## 3. Discussion

Various studies have been investigated to promote the regeneration of periodontal complexes as tooth-supporting structures for natural tooth preservation [1,9,15,22]. However, it is still challenging to secure micron-scaled PDL interfaces against ankylosis (bone fusion to tooth-root surface) and form engineered PDL tissues under spatiotemporal controls of specific fiber orientations, which have oblique or perpendicular angulations to the tooth-root surfaces [23,24]. In particular, angular organizations of principal PDL bundles make significant contributions to modulate biomechanical stimulations during masticatory or occlusal loadings such as resistance or transmission of vertical or intrusive forces [6,8,25]. Therefore, engineered 3D-guiding platforms have been recently investigated for PDL regeneration with angulations such as the soft-lithography method [15,17], the freeze-casting fabrication method [8], or the 3D printing approach [16,18,26]. In particular, the additive manufacturing or 3D printing systems can inherently create topographical artifacts or stair stepping errors on surfaces of created features and the manufacturing resolution is the prominent factor to determine surface qualities. In other words, the high vertical resolution of *z*-axis (or the short interval of stacked layers) in the 3D printing process could provide high printing quality with smooth surfaces or less surface artifacts but the low resolution contributes to create more stair stepping errors and rougher surfaces [16,18]. The previous study reinterpreted the manufacturing artifacts as reproducible surface patterns and the 25.04 μm-microgroove patterns contributed human PDL cells to spatially organize with directionalities, which were associated with the microgroove pattern angles [16]. In the result, angular microgroove pattern surfaces of PDL-guiding architectures regulated orientations of cells at 7 and 21 regardless of the increase of cell populations [16]. At this point, it was critically required to optimize submicron-scaled topographies for highly predictable directionalities of cells and collective cell orientations in the PDL interface between mineralized tissues. This follow-up study was designed for topographical significances to guide and control directionalities of human PDL cells with four different pattern intervals, which were defined during the digital slicing step (Figure 1).

The state-of-the-art approach created four different microgroove patterns with layer thicknesses (or surface microgroove intervals: 25.40 μm; μG-25, 19.05 µm; μG-19, 12.70 µm; μG-12, and 6.35 µm; μG-6), which were fabricated using the digital slicing procedure that could influence PCL scaffold surface patterns (Figure 1). The 3D printed scaffold surfaces were characterized using the SEM for surface morphologies and the confocal microscope for surface topographies (Figure 2). In the qualitative and quantitative analyses, created microgroove pattern intervals on PDL-guiding architectures in PCL scaffolds had no difference from the digital slicing thickness (Figure 2). The lowest resolution (25.04 μm for μG-25) of the printing system facilitated to serve rough surfaces with 25.16 ± 0.97 μm (mean ± standard deviation) microgroove intervals, but the highest resolution (6.35 μm for μG-6) manufactured short microgroove intervals (5.75 ± 1.38 μm) for smooth or high-quality surfaces (Figure 2). Therefore, the 3D wax printing system can significantly offer manufacturing predictability with the microgroove pattern accuracy and reproducibility of designed surface topographies with interval constancies.

After seeding human PDL cells into scaffolds, in vitro cultures were performed for seven days and cell orientations were assessed by staining nuclei and actin filaments in cytoplasm (Figure 3). Although alignments of actin filaments were qualitatively facilitated to identify various orientations or cytoskeletal polarities of single cells following microgroove patterns and their intervals (Figure 3A), it was significantly limited to quantitatively determine angular organizations of collective cells using cytoplasmic components because of the interactive cell-cell junctions, which can be formed during proliferative cell cultures [16]. Because cell angulations could be influenced by nuclear orientations, nuclear morphologies and deformations were specifically measured with the direction of PDL-guiding architectures and analyzed for the correlations between human PDL cells and characterized surfaces of PDL-guiding architectures (Figure 3). Various studies for cell-material interactions have demonstrated that topographical or morphological specificities could be characterized to reflect nuclear deformations, which were associated with cell orientations [21]. Versaevel et al. recently reported the measurements of nuclear angulations could be significantly correlated with cell alignments within micropatterned substrates [21] and Park et al. demonstrated that the nuclear angle measurements to the reference direction could determine spatial organizations of collective cells with specific angles as well as nuclear deformations [16]. As a result, 25.05 μm-distant microgroove intervals (μG-25) showed that over 70% cells of all cells could be angulated similarly to the reference direction, which was designed to guide PDL cells or fibrous tissues, even though the μG-6 group with the highest resolution for the smooth surface quality showed random organizations of cells (Figure 2). In addition to the nuclear angulations, the nuclear shapes were quantitatively analyzed using two different parameters, such as NAR and circularity, which could adjust nuclear conformations and cell orientations to characterize topographies because the cell shapes could be regulated and be dependent on the nuclear morphologies. In Figure 4, NARs and circularities of μG-25 and μG-19 had statistically significant differences from μG-12 and μG-6 groups (*p* < 0.05; Figure 4B,C), so the specific substrates with microgroove patterns and intervals could affect the nuclear deformations and configurations (Figure 4).

The surface quality could be an important factor for 3D printed models, which require high accuracy and precision of manufactured architectures. As such, many studies have attempted to improve surface qualities by developing 3D printing systems for fewer stair stepping artifacts [16,27,28]. In our system, high resolution (6-μm slicing procedure; μG-6) manufacturing could provide smooth surfaces or fewer stair stepping errors, but the μG-6 group showed less controllability and predictability for orientations of fibrous connective tissue cells, which should have specific angulations. Most significantly, topographical artifacts or microgroove patterns at low printing resolution (μG-25) could derive spatiotemporal organizations of fibrous connective (ligamentous) cells and predictably control directionalities of cell collectivity for angulations, which can be determined using the nuclear elongation and deformation on the topographical-characterized substrates to modulate cytoskeletal organizations of actin filament [29,30]. Therefore, the optimized intervals of microgroove patterns should be required to regulate collective cell orientations rather than high surface qualities of PDL-guiding architectures within scaffolds. Our 3D printing system had the limited configuration for the vertical resolution of the additive manufacturing system, but the study can verify the prominent correlation between the microgroove pattern intervals and collective cell alignments as well as anisotropic deformations or angulations of cell nuclei. Including angulated microgroove patterns for cell orientations in the previous study [16], the optimized interval of microgrooves to simply control the printing layer thickness can be a promising approach for 3D platform designs, which can predictably regulate angular orientations of fibrous connective tissues (especially PDLs) in periodontal tissue regeneration.

## 4. Materials and Methods

### 4.1. PDL-Guiding Architecture Design and Biopolymeric Scaffold Fabrication

As the previous study described [16,18], the scaffolds were designed using computer-aided design (CAD) (Solidworks 2020, Dassault Systems SOLIDWORKS Corp., Waltham, MA, USA) and four different layer thickness of designed wax molds were selected at the digital slicing step: 25.40 µm for μG-25, 19.05 µm for μG-19, 12.70 µm for μG-12, and 6.35 µm for μG-6 (Figure 1). After the additive manufacturing process to produce 3D wax molds of four groups by the 3D wax printer (Solidscape 3Z Studio; Solidscape^®^, Inc. Merrimack, NH, USA), which had four typical configurations (25.40 µm, 19.05 µm, 12.70 µm, and 6.35 µm) for the layer-by-layer printing, a build material (blue wax material; Solidscape^®^, Inc.) was selectively removed by ethanol and 25% poly-ε-caprolactone (PCL; MW 43~50 kDa, Polysciences Inc. Warrington, PA USA) solution in 1,4-dioxane was cast into the support material mold (red wax material; Solidscape^®^, Inc.) [16,18]. The PCL-casted wax molds were frozen and then, 1,4-dioxane was extracted using ethanol at −20 °C for 2 days and water at 4 °C for 2 days. Cyclohexane (Sigma-Aldrich, St. Louis, MO, USA) was utilized to dissolve red wax molds at 35–37 °C for 1–2 days and PCL scaffolds were obtained (Figure 1).

### 4.2. Surface Characterizations of Microgroove Patterns on PDL-Guiding Architectures

The morphologies and topographies of patterned surfaces were qualitatively and quantitatively analyzed using a scanning electron microscope (SEM; S-4700 FE-SEM, Hitachi, Tokyo, Japan) at 15 kV (Figure 2A). Using ImageJ software (National Institutes of Health (NIH), Bethesda, MD, USA), the additively manufactured patterns on the surfaces in individual groups (μG-25, μG-19, μG-12, and μG-6) were qualitatively evaluated by creating topographical profiles (Figure 2B). Moreover, experimental groups with four different intervals of microgroove patterns on scaffold surfaces were topographically and spatially characterized using confocal laser scanning microscope (CLSM; Carl Zeiss MicroImaging GmbH, Jena, Germany; Figure 2C).

### 4.3. Cell Orientation and Nuclear Deformation Analyses in In Vitro

The human PDL cells were cultured and incubated in passage 4–6 using the cell growth medium (minimum essential medium alpha; α-MEM) with supplements such as 10% fetal bovine serum (FBS) and antibiotics (100 units/mL penicillin). A total of 1.0 × 10^3^ human PDL cells were seeded into all scaffolds (*n* = 3 per group) and cell-scaffold constructs were incubated for seven days. Fluorescence images were utilized to determine essential difference of cell orientations or cytoskeletal polarities depending on typical intervals of microgroove patterns by staining DAPI (4′,6-diamidino-2-phenylindole, Thermo Fischer Scientific, Waltham, MA, USA) and F-actin staining with phalloidin (Alexa Fluoro^®^ 546 Phalloidin, Life Technologies, Carlsbad, CA, USA) after the cell fixation step. Using DAPI-stained cell nuclei, the nuclear aspect ratio (NAR), circularity (nuclear shape index; NSI), and nuclear orientations to the reference direction (PDL-guiding architecture direction) were measured and calculated by ImageJ software [16,18]. NAR was calculated with the lengths of long and short axes (NAR = long axis/short axis) and NSI was determined with the circularity (or NSI), which could be identified between zero (NSI = 0; a linear shape) and one (NSI = 1; a circular shape) (Figure 4).

### 4.4. Statistical Analyses

All data were calculated and analyzed with the mean ± standard deviation using IBM SPSS Statistics 25 (IBM, Armonk, NY, USA). For statistical assessments of nuclear deformation such as NAR and circularity (or NSI) with four groups (μG-25, μG-19, μG-12, and μG-6), one-way analysis of variance (one-way ANOVA) test with the Bonferroni post-hoc analysis was applied with the α-value set at the 0.05 level of significance.

## 5. Conclusions

In general, the surface quality of scaffolds can be determined by vertical printing resolution during 3D printing process, so the lower layer thickness (higher additive manufacturing resolution) could provide less stair stepping errors or smooth surfaces. However, the follow-up study investigated how specific intervals of microgroove patterns (μG-25), rather than smooth topography with high surface quality of PDL-guiding architectures (μG-6), could guide collective cell alignments with high predictability and controllability. In conclusion, the study demonstrated that 3D printing can provide (1) reproducible 3D platforms with various microgroove patterns, (2) consistent layer intervals to characterize specific topographies with structural accuracy and precision, and (3) angular organizations of ligamentous cells via specific topographies, which can be created by a lower additive manufacturing resolution. The topography optimization to regulate PDL cell orientations can offer significant tissue engineering applications for fibrous connective tissue orientations or biomechanical functioning restorations in musculoskeletal tissue reconstructions.

## Abbreviations

PDL	Periodontal ligament
3D	Three dimensional
μG-25	Microgroove pattern interval with 25.40 μm
μG-19	Microgroove pattern interval with 19.05 μm
μG-12	Microgroove pattern interval with 12.70 μm
μG-6	Microgroove pattern interval with 6.35 μm
PCL	poly-ε-caprolactone
SEM	Scanning electron microscope
DAPI	4′,6-diamidino-2-phenylindole
NAR	Nuclear aspect ratio
NSI	Nuclear shape index
CAD	Computer-aided design
α-MEM	Minimum essential medium alpha
FBS	Fetal bovine serum
ANOVA	Analysis of variance
